# Betulin, a Newly Characterized Compound in *Acacia auriculiformis* Bark, Is a Multi-Target Protein Kinase Inhibitor

**DOI:** 10.3390/molecules26154599

**Published:** 2021-07-29

**Authors:** Augustine A. Ahmadu, Claire Delehouzé, Anas Haruna, Lukman Mustapha, Bilqis A. Lawal, Aniefiok Udobre, Blandine Baratte, Camilla Triscornia, Axelle Autret, Thomas Robert, Jeannette Chloë Bulinski, Morgane Rousselot, Mélanie Simoes Eugénio, Marie-Thérèse Dimanche-Boitrel, Jacobus P. Petzer, Lesetja J. Legoabe, Stéphane Bach

**Affiliations:** 1 Department of Pharmaceutical and Medicinal Chemistry, Faculty of Pharmaceutical Sciences, Kaduna State University, Kaduna 800241, Nigeria; anas.haruna@kasu.edu.ng (A.H.); lukman.mustapha@kasu.edu.ng (L.M.); 2 Department of Pharmaceutical and Medicinal Chemistry, Faculty of Pharmacy, University of Calabar, Calabar 540271, Nigeria; 3 Station Biologique de Roscoff, CNRS, UMR8227, Integrative Biology of Marine Models Laboratory (LBI2M), Sorbonne Université, 29680 Roscoff, France; claire.delehouze@seabelife.com (C.D.); baratte@sb-roscoff.fr (B.B.); camilla.triscornia@gmail.com (C.T.); trobert@sb-roscoff.fr (T.R.); jcb4@cumc.columbia.edu (J.C.B.); 4 Place Georges Teissier, SeaBeLife Biotech, 29680 Roscoff, France; axelle.autret@lekreisker.fr (A.A.); morgane.rousselot@seabelife.com (M.R.); melanie.simoeseugenio@univ-rennes1.fr (M.S.E.); 5 Department of Pharmacognosy and Drug Development, Faculty of Pharmaceutical Sciences, University of Ilorin, Ilorin 240003, Nigeria; lawal.ba@unilorin.edu.ng; 6 Department of Pharmaceutical and Medicinal Chemistry, Faculty of Pharmacy, University of Uyo, Uyo 520003, Nigeria; aniefiokudobre@uniuyo.edu.ng; 7 CNRS, FR2424, Station Biologique de Roscoff, Plateforme de Criblage KISSf (Kinase Inhibitor Specialized Screening Facility), Sorbonne Université, 29680 Roscoff, France; 8 Department of Biological Sciences, Columbia University, New York, NY 10027, USA; 9 Institut de Recherche sur la Santé, l’Environnement et le Travail (IRSET), INSERM UMR 1085, F-35043 Rennes, France; marie-therese.boitrel@univ-rennes1.fr; 10 Biosit UMS 3080, Université de Rennes 1, F-35043 Rennes, France; 11 Centre of Excellence for Pharmaceutical Sciences, North-West University, Private Bag X6001, Potchefstroom 2520, South Africa; Jacques.Petzer@nwu.ac.za (J.P.P.); Lesetja.Legoabe@nwu.ac.za (L.J.L.); 12 Pharmaceutical Chemistry, School of Pharmacy, North-West University, Private Bag X6001, Potchefstroom 2520, South Africa

**Keywords:** triterpenoids, Acacia stem bark, polypharmacology, protein kinase inhibitors

## Abstract

The purpose of this work is to investigate the protein kinase inhibitory activity of constituents from *Acacia auriculiformis* stem bark. Column chromatography and NMR spectroscopy were used to purify and characterize betulin from an ethyl acetate soluble fraction of acacia bark. Betulin, a known inducer of apoptosis, was screened against a panel of 16 disease-related protein kinases. Betulin was shown to inhibit Abelson murine leukemia viral oncogene homolog 1 (ABL1) kinase, casein kinase 1ε (CK1ε), glycogen synthase kinase 3α/β (GSK-3 α/β), Janus kinase 3 (JAK3), NIMA Related Kinase 6 (NEK6), and vascular endothelial growth factor receptor 2 kinase (VEGFR2) with activities in the micromolar range for each. The effect of betulin on the cell viability of doxorubicin-resistant K562R chronic myelogenous leukemia cells was then verified to investigate its putative use as an anti-cancer compound. Betulin was shown to modulate the mitogen-activated protein (MAP) kinase pathway, with activity similar to that of imatinib mesylate, a known ABL1 kinase inhibitor. The interaction of betulin and ABL1 was studied by molecular docking, revealing an interaction of the inhibitor with the ABL1 ATP binding pocket. Together, these data demonstrate that betulin is a multi-target inhibitor of protein kinases, an activity that can contribute to the anticancer properties of the natural compound and to potential treatments for leukemia.

## 1. Introduction

Terrestrial plants are a crucial source of medicines, especially in developing countries. According to the WHO, about 80% of the world’s population depends on plant-derived medicines for their health care [1,2]. Secondary metabolites present in natural extracts purified from plants and microorganisms are known to possess multiple bioactivities (ranging from cytotoxic to cytoprotective) [3]. Accordingly, intensive study has been devoted to the purification and chemical characterization of active constituents that could possibly yield a novel chemical compound suitable for drug development [4,5]. Human protein kinases represent the third largest enzyme class and are responsible for modifying up to one-third of the human proteome. Over 518+ protein kinases, including serine/threonine and tyrosine kinases, are encoded by the human genome [6]. Dysregulation of kinase function (e.g., by hyperactivation, or mutation) plays an important role in many diseases, such as cancer [7], neurodegenerative disorders, inflammation, and diabetes, thereby making protein kinases attractive targets for the pharmaceutical industry [8]. From 20 to 33% of current drug discovery efforts worldwide are focused on the protein kinases [9]. Consequently, the FDA in the United States has already approved 65 small molecule protein kinase inhibitors, as of July 2021 [10,11,12]. At least 18 of these kinase inhibitors inhibit a number of kinases; that is, they are multi-target inhibitors [9]. Metabolites from plants are known to be a rich source of putative protein kinase inhibitors (e.g., flavonoid compounds that function as competitive inhibitors of ATP binding [13]).

The genus *Acacia* belongs to the family *Fabaceae* and includes about 1400 species of trees and shrubs widespread throughout warm and semiarid regions of the world including subtropical and tropical Africa (e.g., Nigeria, Senegal, Egypt, and Mozambique) [14]. Within this vast genus, *Acacia auriculiformis*, commonly referred to as Black Wattle, is an important medicinal plant. The Ibibio community of Niger Delta region in Nigeria uses this plant as antimalarial [15]. Moreover, an infusion of the bark of this plant is used to treat inflammation among the aborigines of Australia [16]. Several Acacia species including Black Wattle are also known to contain components that inhibit tumor growth, and thus understanding the mechanism for the reported activity is of great interest [17]. In addition, the antimutagenic and chemoprotective activities of *Acacia auriculiformis*, particularly the tannins contained in the bark, as well as the ability of its ethyl acetate and acetone extracts to scavenge free radicals have been reported [18,19,20]. Recent publications have shown the purification of one new triterpenoid trisaccharide and three new triterpenoids, and have demonstrated antimicrobial activity of acaciaside a and acaciaside b [21,22]. The mosquito larvicidal activity of the fruit extracts and the protein kinase inhibitory activity of a tetrahydroxy flavone isolated from the stem bark of *Acacia auriculiformis* have also been reported [23,24].

In the present study, we report the isolation of the triterpenoid betulin and the investigation of this compound’s activity against a panel of disease-related kinases. We also demonstrate the effect of betulin on the viability of doxorubicin-resistant and -sensitive human leukemia cell lines.

## 2. Results

### 2.1. Purification of Betulin from Acacia Auriculiformis Stem Bark and Evaluation of Its Biological Activity against Disease-Related Protein Kinases

**Figure 1:**
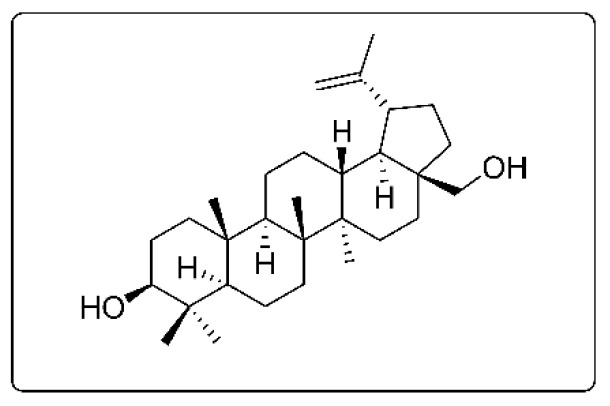
Chemical structure of betulin (3-lup-20(29)-ene-3*β*,28-diol, molecular weight = 442.72 g/mol).

Preliminary kinase-based screening was carried out using *Acacia auriculiformis* stem bark extracts, and it was discovered that the ethyl acetate soluble fraction was the most active among the three fractions investigated, namely chloroform, ethyl acetate, and N-butanol [23]. Chromatographic purification of the compound(s) that might be responsible for the kinase inhibition from the ethyl acetate soluble fraction led to the isolation of a compound as a white amorphous solid. This compound displayed spectral properties (^1^H and ^13^C, see Figures S1–S6 for ^13^C-NMR (DEPT) and proton NMR spectra of the betulin purified from *Acacia*) consistent with literature data for betulin (3-lup-20(29)-ene-3*β*,28-diol) [25]. *Acacia auriculiformis* stem bark was found to contain about 0.002% of betulin by dry weight. The chemical structure of betulin is depicted on Figure 1.

As triterpenoids have already been shown to inhibit protein kinases, we tested the inhibitory effect of betulin on a panel of protein kinases (PKs). Eight disease-related human PKs were tested including cyclin-dependent kinases (CDK5/p25 and CDK9/CyclinT), Haspin, proto-oncogene proviral integration site for moloney murine leukemia virus-1 (Pim1), glycogen synthase kinase-3 beta (GSK-3β), casein kinase 1 epsilon (CK1ε), Janus kinase 3 (JAK3), and Abelson murine leukemia viral oncogene homolog 1 (ABL1). Table 1 shows the results of the primary screening. Betulin showed weak activity against Pim1; i.e., at 10 µg/mL, it showed only 10% inhibition of kinase activity. This contrasted markedly with ABL1, whose kinase activity was inhibited by 79%. JAK3 and GSK-3β also showed inhibition by betulin.

**Figure 2:**
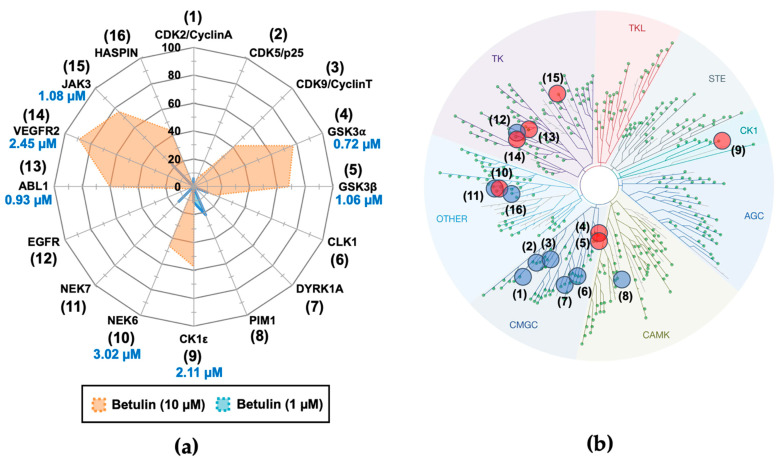
Selectivity of betulin against a panel of 16 disease-related protein kinases. (**a**) The value reported on the Kiviat chart is mean (n = 2) expressed in % of inhibition, compared with a DMSO control. The IC_50_ values in µM are listed below the name of each tested kinase. As betulin was not abundant in Acacia bark, we used a commercially-available betulin, provided by Sigma (reference #B9757). All protein kinases used here are human except DYRK1A (*Rattus norvegicus*) and CLK1 (*Mus musculus*). (**b**) The targets used here are selected from the human kinome as represented in the panel by blue dots on the circular tree. Red dots indicate that the kinase is inhibited by betulin. This image was generated using the TREEspot™ Software Tool (Eurofins DiscoverX Corporation, Fremont, CA, USA) and reprinted with permission from KINOMEscan^®^, a division of Eurofins DiscoverX Corporation (© DiscoverX Corporation 2010). The codes reported on this figure indicate the subclasses of protein kinases: CMGC for CDKs, MAP kinases, GSK, and CDK-like kinases; AGC for protein Kinase A, C, and G families (PKA, PKC, PKG); CAMK for Ca^2+^/calmodulin-dependent protein kinases; CK1, Cell/Casein Kinase 1; STE, STE Kinases (Homologs of yeast STErile kinases); TKL, Tyrosine Kinases-Like; and TK, Tyrosine Kinases.

**Figure 3:**
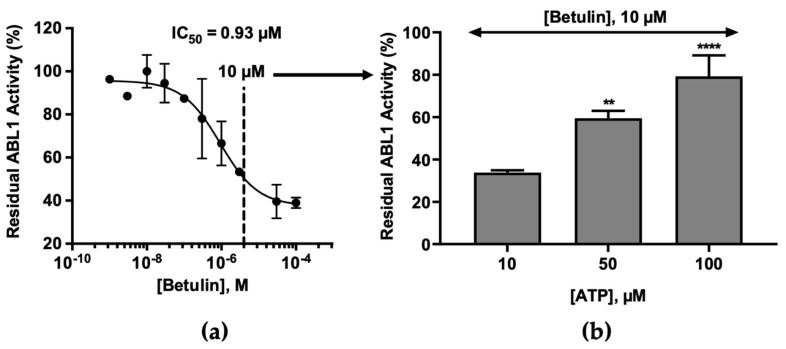
Effect of ATP on the inhibition of ABL1 kinase by betulin. (**a**) The IC_50_ value of betulin against human ABL1 kinase was determined from the dose-response curve using GraphPad PRISM Software. ATP concentration used in the kinase assays was 10 µM (values are means, n = 2). (**b**) We selected 10 µM of betulin as fixed concentration of inhibitor (approximately 10 times the IC_50_ value) and measured the inhibition by betulin at different ATP concentrations by ADP-Glo luminescent assay: 10, 50, and 100 µM. Data represent the mean (n = 4) ± SD expressed in % of residual activity, compared with a DMSO control. ** *p* < 0.01 vs. ATP [10 µM], **** *p* < 0.0001 vs. ATP [10 µM].

Betulin was next tested against a larger panel of 16 protein kinases, as reported in Figure 2, using a range of betulin concentrations. IC_50_ values were determined from the dose-response curves for the target kinases most potently inhibited by betulin (displaying more than ~45% of inhibition at 10 µM of betulin). Results in Figure 2 revealed that betulin gave the highest inhibitory activity against GSK-3α, with IC_50_ of 0.72 µM; followed by ABL1, with IC_50_ of 0.93 µM (see Figure 3a for dose-response curve for ABL1); GSK-3β, with IC_50_ of 1.06 µM; JAK3 kinase with IC_50_ of 1.08 µM; CK1ε, with IC_50_ of 2.11 µM; vascular endothelial growth factor receptor 2 (VEGFR2), with IC_50_ of 2.45 µM; and NIMA Related Kinase 6 (NEK6), with IC_50_ of 3.02 µM. Given these results and the well-established role of BCR-ABL1 in chronic myelogenous leukemia (CML), we next focused our work on the inhibition of ABL1 by betulin.

### 2.2. Molecular Mechanism of ABL1 Inhibition by Betulin

To test the hypothesis that kinase inhibition by betulin might be the driver of its cellular effects, we explored the binding mode of betulin to ABL1, using ATP competition assays. Accordingly, we measured % of maximal activity (relative to a DMSO control) remaining in the presence of betulin, at ATP concentrations of 10, 50, and 100 µM. As shown in Figure 3, the results obtained strongly suggest competitive inhibition of ATP-binding to ABL1 by betulin. The inhibition of the ABL1 activity by 10 µM betulin was significantly decreased in the presence of a high concentration of ATP (100 µM). We note here that other triterpenoids, for example those extracted from the dry infructescences of *Liquidambaris Fructus* (also called Lu Lu Tong when used in Traditional Chinese medicine to treat some breast disease) have also been implicated as putative ATP competitors [26].

### 2.3. Molecular Modeling of the ABL1-Betulin Complex

**Figure 4:**
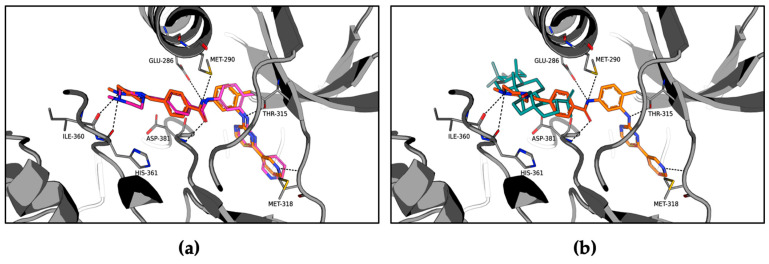
In silico docking analysis of the interaction between the ATP binding site of ABL1 and imatinib or betulin. (**a**) The binding orientation and interactions of imatinib with the ABL1 tyrosine kinase as exhibited in the crystal structure (orange, PDB code: 1IEP) compared to the orientation predicted with molecular docking (magenta). (**b**) The binding orientations and interactions of imatinib (orange) and betulin (teal) with the ABL1 tyrosine kinase ATP binding site as predicted by molecular docking.

To gain further insight, we investigated the interaction of betulin with the ATP binding site of ABL1 tyrosine kinase by molecular docking. To accomplish this, we used the crystal structure of ABL1 tyrosine kinase complexed with the established inhibitor, imatinib, as an adduct, and carried out docking with Discovery Studio 3.1 and AutoDock Vina [27,28] software. The accuracy of the docking procedure was evaluated by docking imatinib back into its established binding site. The root mean square deviation (RMSD) of the highest-ranked orientation from the position of the imatinib in the crystal structure was found to be 1.01 Å (Figure 4). We note that RMSD values <1.5 Å are considered to indicate successful molecular docking [29].

The results show that betulin fits within the ATP binding site of ABL1 tyrosine kinase, in a position that overlaps with the methylpiperazine ring of imatinib (Figure 4). The positioning of betulin is similar to that predicted recently for a pentacyclic triterpenoid gypsogenin derivative in a recent report [30]. In contrast to imatinib, the large size of the betulin molecule sterically hinders it from binding deep within the binding pocket. The secondary alcohol extends towards the exterior of the protein while the hydroxymethyl and vinyl substituents are directed towards the interior of the protein. While imatinib undergoes extensive hydrogen bonding, the lipophilic structure of betulin undergoes mostly van der Waals interactions with the surrounding amino acid residues (e.g., Glu286, Met290, Ile293, Val298, Leu354, Ile360, His361, Arg362, and Asp381).

This computational approach suggests that betulin is located far from amino acid residue Thr315, a residue of particular interest as the T315I mutation of ABL1 kinase attenuates inhibition by imatinib. To test this notion, we assayed betulin effects on ABL1 kinase, bearing the T315I mutation in its kinase domain. As shown in Figure S7, betulin at a concentration of 1µM, i.e., a dose similar to the IC_50_ value of betulin against wild type ABL1, retains its capacity to inhibit the enzymatic activity of T315I mutant of ABL1. As expected, inhibition of T315I mutant of ABL1 kinase by imatinib is strongly reduced. These results support the putative binding mode of betulin determined by molecular docking (Figure 4).

### 2.4. Betulin Selectively Inhibits Proliferation of Human Leukemic Cells

**Figure 5:**
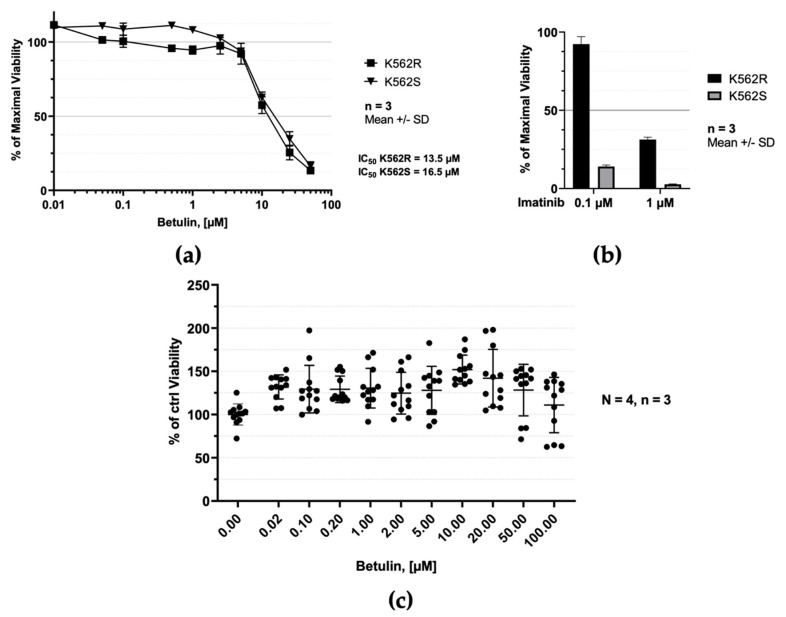
In vitro effects of betulin on the viability of K562 chronic myelogenous leukemia (CML) cells and human peripheral blood lymphocytes (hPBLs). (**a**) The viability of K562S and K562R CML cell lines, which are respectively sensitive (S) and resistant (R) to treatment with doxorubicin, was studied with the MTS assay. Cell viability was measured after 48 h exposure to increasing doses of betulin. The IC_50_ values were determined from the dose-response curves using GraphPad PRISM Software. Data are given as mean ± SD (n = 3) expressed in % of maximal viability (normal cells treated with the identical quantity of vehicle, DMSO, only). (**b**) The viability of K562S and K562R CML cell lines was measured after 48 h exposure to 0.1 or 1 µM of imatinib mesylate. Data are provided as mean ± SD (n = 3) expressed in % of maximal viability (relative to DMSO control, as in (**a**)). (**c**) Cell viability 48 h after treatment with increasing concentrations of betulin (0.02– 100 µM), measured with MTS assay, showing the toxicity of betulin towards human PBL. Data are expressed as mean ± SD (N = 4, n = 3).

The anticancer and chemoprotective potential of betulin has already been reported (see [31] for a table reporting the in vitro antiproliferative effect of betulin on >40 cancer cell lines). Because betulin inhibits ABL1, a kinase shown to cause chronic myelogenous leukemia (CML) when deregulated by fusion with BCR, we next used the human K562 CML cell line to test the effects of betulin in cultured cells (Figure 5a). Although the effects of betulin on K562 cells has been reported in the literature, the results seem to be quite variable: for half-maximal inhibition of cell growth, IC_50_ from 14.5 µM to >200 µM has been reported [31]. Using cell viability to evaluate the betulin activity in K562 cells, we determined that: (i) betulin decreased the viability of K562 leukemic cells in a 48-h assay, with an IC_50_ of 16.5 µM; (ii) leukemia cells resistant to treatment with doxorubicin (a chemotherapeutic drug marketed as Adriamycin^®^, see Figure S8) were equally sensitive to treatment with betulin (IC_50_ of 13.5 µM) as doxorubicin-sensitive cells; in contrast, the doxorubicin-resistant cells were less sensitive to imatinib mesylate (Figure 5b). Note here that the effects of betulin on cell viability were not significantly altered when cells were treated with 20 µM z-VAD-fmk, a pan-inhibitor of caspases (Figure S9).

The efficacy of cancer chemotherapy is critically dependent upon tumor cell selectivity. We next tested the effect of betulin on human peripheral blood lymphocytes (hPBLs) purified from four healthy donors. As shown in Figure 5c, treatment with betulin ≤ 100 µM did not induce a significant decrease of the viability of hPBLs. This result indicates an acceptable level of selectivity of betulin against cancer cells.

### 2.5. Effects of Betulin on the MAPK/ERK Signaling Pathway

**Figure 6:**
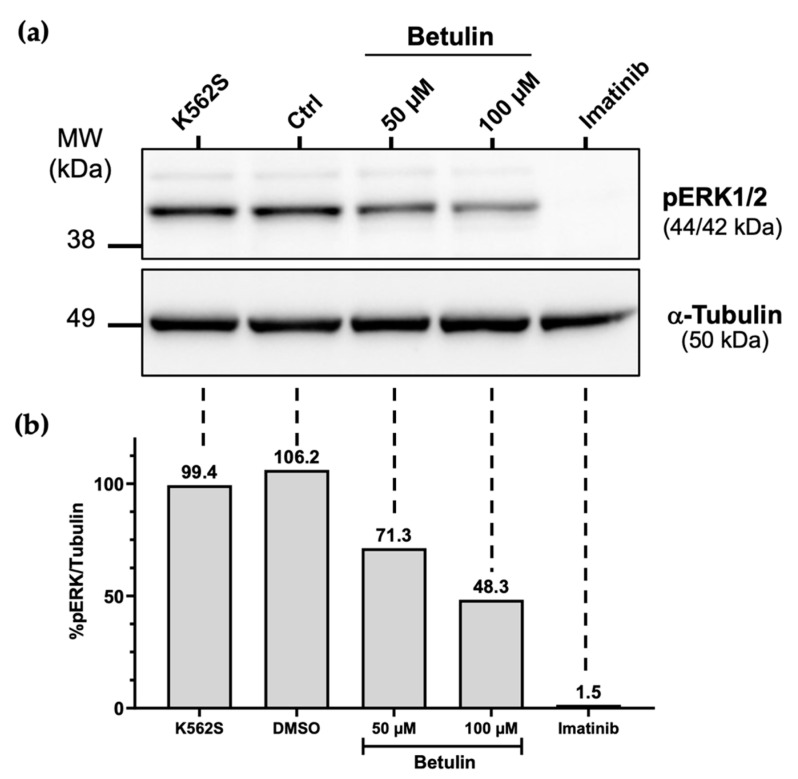
Effects of betulin on extracellular signal-regulated kinase (ERK) signaling. (**a**) K562S CML cells that were untreated (K562S) or treated with 1% DMSO, 50 or 100 µM betulin, or 20 µM of imatinib mesylate for 6 h were subjected to an immunoblot analysis as described in the Methods section. Briefly, extracts of treated K562S cells were analyzed by SDS-PAGE, followed by Western blotting with antibodies directed against phospho-ERK1/2 (Thr202/Tyr204) and α-Tubulin (as a loading control). (**b**) The open source image processing program “ImageJ” was used to quantify the intensity of each band.

The chimeric protein BCR-ABL1 was previously shown to drive neoplastic transformation of hematopoietic stem cells in chronic myelogenous leukemia [32]. ABL1 is the kinase portion of the BCR-ABL1 oncogene. In the BCR-ABL1 fusion protein, ABL1 tyrosine kinase activity is constitutively activated to interact in various signaling pathways including most notably the mitogen activated protein kinase (MAPK)/extracellular-signal-regulated kinase (ERK) pathway that increases cellular proliferation [30]. The K562 CML cell line is known to express the bcr-abl fusion gene [33]. Accordingly we examined phosphorylation of ERK kinases in doxorubicin-sensitive K562 (K562S) cells treated with 50 or 100 µM betulin or 20 µM imatinib mesylate (the latter dose has already been shown to be effective on ERK phosphorylation [30]). All of the treatments tested were shown to decrease the viability of K562S cells after a 48 h treatment (see Figure 5a,b). In Figure 6, we prepared extracts of treated cells after 6 h treatment, so we decided to use a higher dose of each compound compared to that necessary to affect cell viability (~3–6 times more than the IC_50_ of betulin reported in Figure 5a). This strategy was employed to obtain a test for effects on the signaling pathway at the shorter treatment interval. We conducted immunoblot analysis on the protein extracts using anti-phospho-ERK1/2 antibody. As shown in Figure 6 and Figure S10, betulin treatment of cells inhibited the phosphorylation of ERK in a dose-dependent manner. As a control, imatinib mesylate showed a stronger effect and almost completely abrogated the phosphorylation of ERK (Figure 6b). This result demonstrates that betulin markedly decreases the downstream signaling of BCR-ABL oncoprotein.

## 3. Discussion

Betulin is a pentacyclic triterpenoid of lupane type found in plants, and it is naturally abundant in many species of trees in northern Europe. In some birch tree species, the quantity of betulin can be over 50% of the dry weight of the bark [31,34]. In this study, we isolated betulin for the first time from *Acacia auriculiformis* stem bark, in which it is four orders of magnitude less abundant.

Betulin and its derivatives were intensively studied and found to exhibit a broad spectrum of pharmacological activities, including anti-cancer, anti-viral, anti-microbial, anti-inflammatory, and anti-fibrotic effects [35,36]. Moreover, betulin-containing extracts from birch bark were formulated as an oleogel (Episalvan^®^, also known as Oleogel-S10), in which betulin was shown to be the active pharmaceutical ingredient. Oleogel-S10 was approved in 2016 by the European Medicines Agency (EMA) for treatment of partial thickness wounds in adults and was in a Phase III efficacy and safety study since 2017, in patients with inherited epidermolysis bullosa (NCT03068780) [37]. Despite growing interest in therapeutic use of betulin, notably for cancer treatment, the molecular mechanism of action of betulin is not well understood [31].

As previously described, triterpenoids were shown to inhibit various protein kinases: e.g., ursolic acid has been reported to inhibit tyrosine kinase activity [38,39], and plant-derived pentacyclic triterpenoid gypsogenin and derivatives were reported by Ciftci et al. to show activity against myelogenous leukemia by virtue of their inhibition of ABL1 kinase [30]. We thus tested betulin against a panel of disease-related human kinases. Betulin was shown to inhibit several kinases in the panel, with activity in the micromolar range, including ABL1, CK1ε, GSK-3α/β, JAK3, NEK6, and VEGFR2.

Especially notable amongst our results is the inhibitory activity of betulin against ABL1 kinase (IC_50_ of 0.93 µM). ABL1 kinase, a member of the Abelson kinase family that also includes ABL2, has been implicated in cancer, particularly in hematological malignancies such as acute myeloid leukemia (AML), chronic myeloid leukemia (CML), and lymphoblastic leukemia [3]. At present, ABL1 kinase is among the most common drug targets of approved therapeutic kinase inhibitors (ABL1 is the target of five molecular entities approved by FDA for cancer therapy) [12,40]. The activated chimeric BCR-ABL tyrosine kinase is the key biochemical defect that causes Philadelphia chromosome-positive chronic myeloid leukemia (Ph+ CML) [40]. In our study, we showed that betulin inhibits the enzymatic activity of ABL1 and perturbs the MAPK/ERK signaling pathway in chronic myelogenous leukemia cells. Since the antineoplastic mechanism of action of betulin is not yet known, our results raise the possibility that its antitumor mechanism may be at least partially explained by its inhibition of kinases. In future studies, we will increase the panel of kinases, in order to explore more deeply the kinome and to test whether any other kinases are more potently inhibited by betulin than those we found already.

Multidrug resistance (MDR) mediated by the drug efflux protein, P-glycoprotein (P-gp), is one of the major obstacles to successful cancer chemotherapy [41]. As an example, cancer cells use P-gp to escape cell death induced by doxorubicin chemotherapeutic agent [42]. We showed here that contrary to doxorubicin, K562 doxorubicin-resistant cells retained an undiminished sensitivity to betulin. This result indicates that betulin is probably not a substrate for the P-glycoprotein, a crucial factor in considering its potential as part of a treatment strategy to combat human MDR cancers.

The results obtained in this study shed light on a putative mechanism of action of betulin that may drive its known effects on cancer cells. Indeed, betulin was shown to have a multi-pharmacological profile, affecting notably ABL1, JAK3, and GSK-3α/β. These results support the notion that betulin could be used alone or in combination with other anticancer drugs as a putative natural product-based therapeutic for the treatment of haematological malignancies caused by deregulation of protein kinases.

## 4. Materials and Methods

### 4.1. Reagents

Betulin (Lup-20(29)-ene-3*β*,28-diol, product reference B9757, purity ≥ 98%) was from Sigma-Aldrich (St. Louis, MO, USA). Imatinib mesylate (Gleevec^TM^, product reference S1026) was from Selleckchem (Houston, TX, USA). z-VAD-fmk was obtained from Enzo Life Sciences (Villeurbanne, France). Doxorubicin was obtained from Teva Pharmaceutical (Petah Tikva, Israel). Stock solutions of all drugs tested were prepared in dimethyl sulfoxide (DMSO).

Note here that the poor solubility in water of betulin was reported in the literature: betulin was shown to be soluble to only 0.08 μg/mL (0.18 µM) [43,44] and had a high predicted Octanol-Water Partition Coefficient (Log*P*) of 9.01 (data from ChemSpider database, Royal Society of Chemistry). Consequently, in this study, betulin was solubilized in DMSO at 10 mM final concentration just before use, avoiding storage at −20 °C. For cell-based assays, dilutions were performed in culture media to achieve 0.5–1% DMSO final concentration. For kinase assays, dilutions were prepared in water to reach 1% DMSO final concentration.

### 4.2. Cell Lines and Culture

K562 (ATCC^®^, CCL-243, described here as K562S to indicate sensitivity to doxorubicin), a human chronic myelogenous leukemia cell line, was obtained from American Type Culture Collection (Manassas, VA, USA). A doxorubicin-resistant cell line (K562R, also known as K562/Adr) was kindly provided by the IRSET institute (Research Institute for Environmental and Occupational Health, INSERM, University of Rennes 1, France). The cells were maintained at 37 °C and 5% CO_2_ in Gibco™ 1640 Roswell Park Memorial Institute (RPMI-1640) medium containing 10% fetal bovine serum (FBS) (Life Technologies^TM^, Thermo Fisher Scientific, Waltham, MA, USA).

Peripheral blood mononuclear cells (PBMCs) were isolated by Ficoll gradient centrifugation from blood buffy coats of healthy donors, provided by the Etablissement Français du Sang (EFS). The research protocol was conducted under French legal guidelines. After separation of monocytes by 1 h adhesion step, non-adherent PBMCs (peripheral blood lymphocytes, PBL) were harvested. PBL were cultured in RPMI 1640 medium (Gibco, Life technologies, Carlsbad, CA, USA) supplemented with 10% decomplemented fetal bovine serum (CVFSVF00–01, Eurobio), penicillin (100 IU/mL), and streptomycin (100 μg/mL) (15140–122, Gibco). All cells were cultured at 37 °C with 5% CO_2_.

### 4.3. Purification of Natural Products from Plant Material

Plant material (consisting of bark) was collected in Samaru-Zaria, Nigeria in September, 2018, and was identified by U.S Gallah, the plant taxonomist of Biological Sciences Department, Kaduna State University, where a voucher specimen (number 1292) was deposited in the herbarium. All protocols involving the collection and use of plant material adhered to relevant ethical guidelines. Air-dried, pulverized bark was extracted with 70 % ethanol at room temperature for 7 days. The combined ethanol extract was concentrated using a rotary evaporator to give a semi-solid mass (45 g). Thirty-two grams of the crude extract was suspended in 100 mL of water and partitioned with 5 × 300 mL of ethyl acetate, and 5 × 300 mL of n-butanol was used to yield 3.6 g and 2.3 g of ethyl acetate- and n-butanol-soluble fractions respectively. A portion of the ethyl acetate-soluble fraction (2.4 g) was packed into a column of silica gel G (200–400 mesh, Silicycle, 5 cm × 50 cm) and eluted first with 100% dichloromethane and then with a stepwise gradient of dichloromethane and methanol mixtures, as follows: 99:1, 98:2, 97:3, 96:4, 95:5, 90:10, 80:20, 60:40, 50:50, 30:70, 10:90, and 100% methanol. The progress of elution was monitored by thin layer chromatography (carried out on pre-coated silica gel TLC plates aluminum backed (Silicycle) using the solvent system ethyl acetate:dichloromethane:methanol:water (15:8:4:1 and 6:4:4:1, respectively). Fractions eluted with 2% methanol in dichloromethane were further purified using Sephadex LH-20 (Pharmacia), eluted with methanol to give natural product betulin, yielding a white solid. The identification of betulin was performed by NMR spectroscopy, carried out on a Bruker Avance NMR spectrophotometer (500 MH_Z_ ^1^H, and 125 MH_Z_ ^13^C).

### 4.4. Protein Kinase Assays

Kinase enzymatic activities were assayed in 384-well plates using the ADP-Glo^TM^ assay kit, following the recommendations of the manufacturer (Promega, Madison, WI). Controls were performed in appropriate dilutions of dimethyl sulfoxide (DMSO). Kinase activities, measured in the presence of 10 µM ATP, are expressed as percentage of maximal activity, i.e., measured in the absence of inhibitor. In order to determine the half-maximal inhibitory concentration (IC_50_), the assays were performed in duplicate in the absence or presence of increasing doses of the tested compounds. Data were analyzed using GraphPad PRISM (GraphPad Software, San Diego, CA, USA) software to fit a sigmoïdal curve that allowed determination of the IC_50_ values. The experimental conditions used for measuring kinase activities are comprehensively described in Ibrahim et al. [45]. Human recombinant ABL1 mutant (T315I) was purchased from Promega (Catalog # V5320) and tested following the recommendations of the manufacturer (Promega, Madison, WI, USA).

### 4.5. Molecular Docking

Molecular docking simulations were carried out with Discovery Studio 3.1 (Accelrys) and AutoDock Vina software [27]. The crystal structure of ABL1 tyrosine kinase complexed with imatinib was obtained from the Brookhaven protein data bank (PDB code: 1IEP) [28]. The protein was prepared and protonated for docking in Discovery Studio with the ‘prepare protein’ function. The pKa values and protonation states of the ionizable amino acids were subsequently calculated at pH 7.4, and the protein model was typed with the Momany and Rone CHARMm forcefield. A fixed atom constraint was applied to the backbone and the protein was energy minimized with the Smart Minimiser algorithm (50,000 steps maximum) using the implicit generalised Born solvation model with molecular volume. Discovery studio was used to construct structures for betulin and imatinib, which were submitted to the ‘prepare ligands’ protocol. The co-crystallised ligand and water molecules were removed from the protein model, and AutoDock Vina was used for the docking. The highest-ranked solution of each ligand was further refined with the Smart Minimizer algorithm. Illustrations were prepared with the PyMOL molecular graphics system [46].

### 4.6. Drug Treatment and Cell Viability Assay

Cell viability was evaluated with the MTS (3-(4,5-dimethylthiazol-2-yl)-5-(3-carboxymethoxyphenyl)-2-(4-sulfophenyl)-2H-tetrazolium) reduction kit, (CellTiter 96 Aqueous Non-Radioactive Cell Proliferation Assay, Promega, Fitchburg, WI, USA), according to the instructions of the manufacturer. Cell viability (% of maximal viability) was quantified following 48 h exposure of human cells (PBLs, human K562S and K562R myelogenous leukaemia cells) to the tested doses of betulin, doxorubicin, or imatinib mesylate (the latter used as a positive control, as an established inhibitor of ABL1 kinase). Further details on experimental conditions used can be found in Delehouzé et al. 2017 [47].

### 4.7. Immunoblot Analysis and Antibodies

K562S cells were treated with 20 µM of imatinib mesylate or 50 and 100 µM of betulin for 6 h. The concentration of DMSO in the culture medium was 1%. Cell lysates were prepared by sonication of the cells in homogenization buffer with 0.5% of nonidet P-40 non-ionic detergent (NP-40) as described previously [48]. SDS-PAGE and immunoblotting were performed following standard procedures. Anti-α-tubulin antibody (clone B512, T5168, 1:5000) was purchased from Sigma Aldrich (St. Louis, MO, USA). The anti-phospho-p44/42 MAPK (#28733–1-AP, 1:2000) was purchased from Proteintech^®^ (Rosemont, IL, USA). This rabbit polyclonal antibody detects p44 and p42 MAPK when doubly phosphorylated (at Thr202 and Tyr204 of p44 and at Thr185 and Tyr187 of p42 MAPK).

## Figures and Tables

**Table 1:** Primary screening of betulin purified from *Acacia auriculiformis* against a panel of eight disease-related human protein kinases.

Betulin ^1^ Tested at:	CDK5/ p25	CDK9/ CyclinT	Haspin	Pim1	GSK-3β	CK1ε	JAK3	ABL1
**10 µg/mL**	99	60	91	90	30	48	25	21
**1 µg/mL**	94	88	72	≥100	38	53	28	36

^1^ Data in the Table represent the % of kinase activity that remained following treatment of each kinase with 10 or 1 µg/mL of betulin, normalized to control (that is, kinase activity in the presence of the vehicle, DMSO, only). ATP concentration used in the kinase assays was 10 µM, and values given represent mean (n = 2). A value ≥100 indicates that the compound does not detectably inhibit the enzymatic activity at the tested concentration.

## Data Availability

Not applicable.

## Supplementary Materials

The following are available online, Figure S1. Proton NMR of betulin in deuterated chloroform (CDCl_3_); Figure S2. Proton NMR spectrum of betulin (Expansion 1); Figure S3. Proton NMR spectrum of betulin (Expansion 2); Figure S4. ^13^C-NMR (DEPT) spectrum of betulin; Figure S5. ^13^C-NMR (DEPT) spectrum of betulin (Expansion 1); Figure S6. ^13^C-NMR (DEPT) spectrum of betulin (Expansion 2); Figure S7. Effect of the T315I ABL1 kinase domain mutation on the inhibition by betulin. Figure S8. In vitro effect of doxorubicin on the cell viability of K562 chronic myelogenous leukemia (CML) cells; Figure S9. The effect of treatment with pan caspase inhibitor z-VAD-fmk on the phenotype induced by betulin; Figure S10. Effects of betulin on extracellular signal-regulated kinases (ERK) signaling.

## Sample Availability

Betulin can be purchased from Sigma-Aldrich (St. Louis, MO, USA) under the reference B9757.
