# Supplementary material for: Betulin, a Newly Characterized Compound in Acacia auriculiformis Bark, Is a Multi-Target Protein Kinase Inhibitor
Source: Molecules. 2021 Jul 29;26(15):4599. doi: 10.3390/molecules26154599 (PMC8347092; doi:10.3390/molecules26154599)
Supplement: Supplementary file 1 [file molecules-26-04599-s001.zip › molecules-1266268-supplementary.pdf]

Supplementary Materials

Betulin, a newly characterized compound in *Acacia auriculiformis* bark, is a multi-target protein kinase inhibitor

Augustine A. Ahmadu[*,,***,1], Claire Delehouzé[2,3], Anas Haruna[1], Lukman Mustapha[1], Bilqis A. Lawal[4], Aniefiok Udobre[5], Blandine Baratte[2,6], Camilla Triscornia[2], Axelle Autret[3], Thomas Robert[2,6], Jeannette Chloë Bulinski[2,7], Morgane Rousselot[3], Mélanie Simoes Eugénio[3,8], Marie-Thérèse Dimanche-Boitrel[8,9], Jacobus P. Petzer[10,11], Lesetja J. Legoabe[10] and Stéphane Bach[**,***,2,6,10]**

[1] Department of Pharmaceutical and Medicinal Chemistry, Faculty of Pharmaceutical Sciences, Kaduna State University, Nigeria; ahmadu2001@yahoo.com; anas.haruna@kasu.edu.ng; lukman.mustapha@kasu.edu.ng

[2] Sorbonne Université, CNRS, UMR8227, Integrative Biology of Marine Models Laboratory (LBI2M), Station Biologique de Roscoff, 29680 Roscoff, France; baratte@sb-roscoff.fr; camilla.triscornia@gmail.com; trobert@sb-roscoff.fr; bach@sb-roscoff.fr

[3] SeaBeLife Biotech, Place Georges Teissier, 29680, Roscoff, France; claire.delehouze@seabelife.com; axelle.autret1@gmail.com; morgane.rousselot@seabelife.com; melanie.simoeseugenio@univ-rennes1.fr

[4] Department of Pharmacognosy and Drug Development, Faculty of Pharmaceutical Sciences, University of Ilorin, Ilorin, Nigeria; lawal.ba@unilorin.edu.ng

[5] Department of Pharmaceutical and Medicinal Chemistry, Faculty of Pharmacy, University of Uyo, Nigeria; aniefiokudobre@uniuyo.edu.ng

[6] Sorbonne Université, CNRS, FR2424, Plateforme de criblage KISSf (*Kinase Inhibitor Specialized Screening facility*), Station Biologique de Roscoff, 29680 Roscoff, France

[7] Department of Biological Sciences, Columbia University, New York, NY 10027, USA; jcb4@cumc.columbia.edu

[8] INSERM UMR 1085, Institut de Recherche sur la Santé, l'Environnement et le Travail (IRSET), F-35043 Rennes, France; marie-therese.boitrel@univ-rennes1.fr

[9] Biosit UMS 3080, Université de Rennes 1, F-35043 Rennes, France

[10] Centre of Excellence for Pharmaceutical Sciences, North-West University, Private Bag X6001, Potchefstroom 2520, South Africa; Jacques.Petzer@nwu.ac.za; Lesetja.Legoabe@nwu.ac.za

[11] Pharmaceutical Chemistry, School of Pharmacy, North-West University, Private Bag X6001, Potchefstroom 2520, South Africa

* Current address: Department of Pharmaceutical and Medicinal Chemistry, Faculty of Pharmacy, University of Calabar, Nigeria.

** Correspondence: ahmadu2001@yahoo.com; Tel.: +234-80-37-03-35-05 (AA); bach@sb-roscoff.fr; Tel.: +33 2 98 29 23 91 (S.B.).

*** These authors share senior authorship.

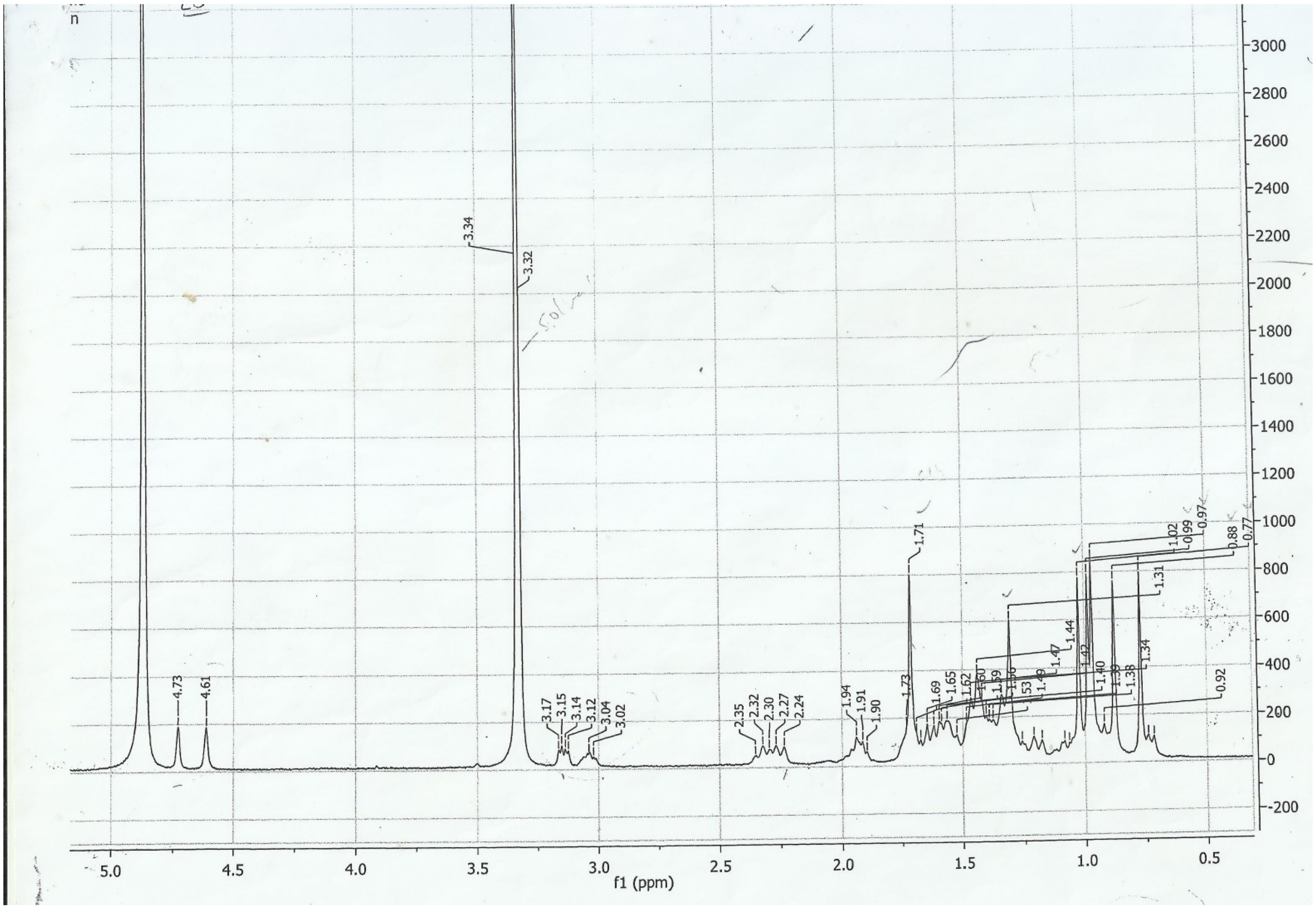

Figure S1. Proton NMR of betulin in deuterated chloroform ($CDCl_3$).

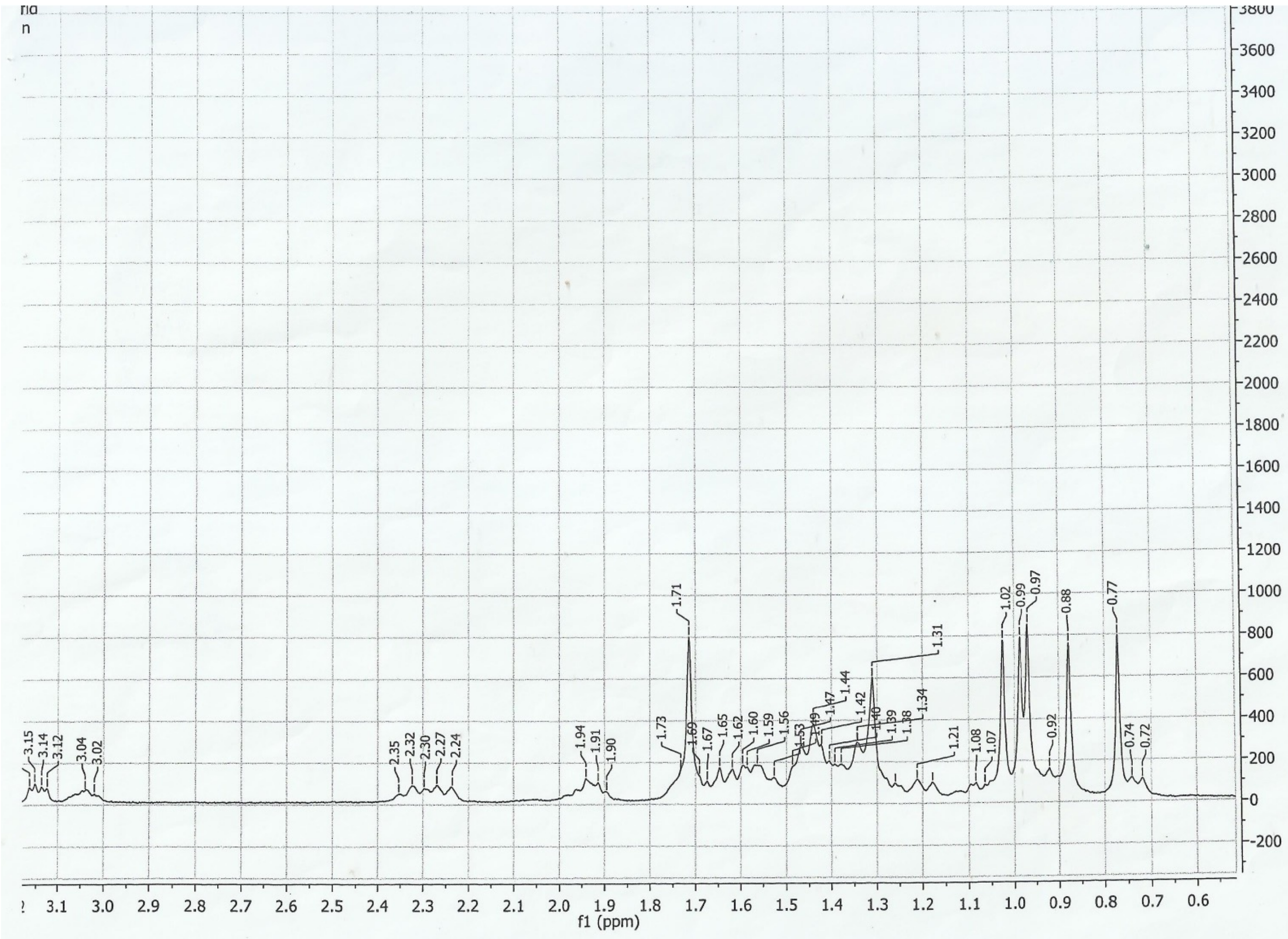

Figure S2. Proton NMR spectrum of betulin (Expansion 1).

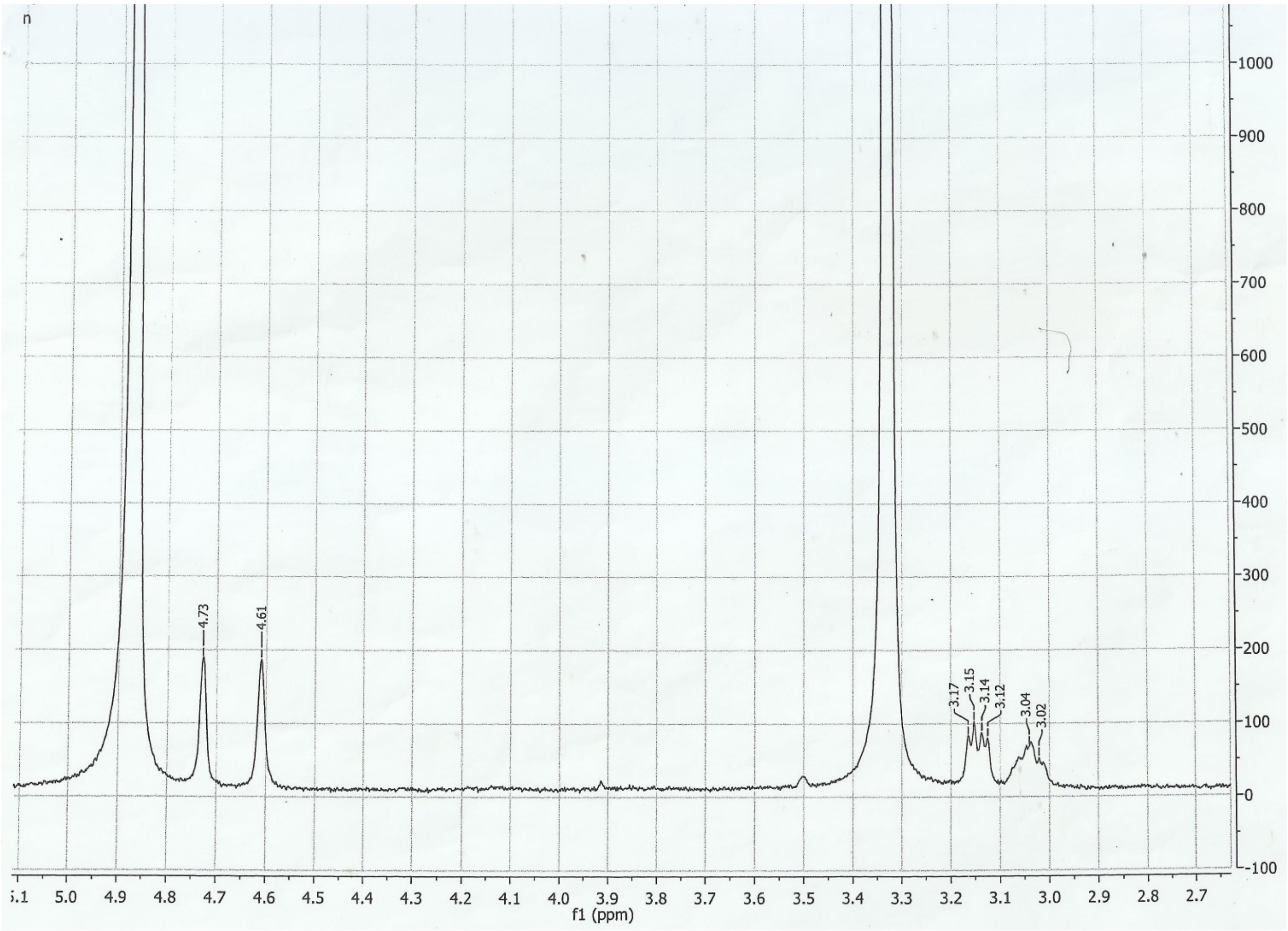

Figure S3. Proton NMR spectrum of betulin (Expansion 2).

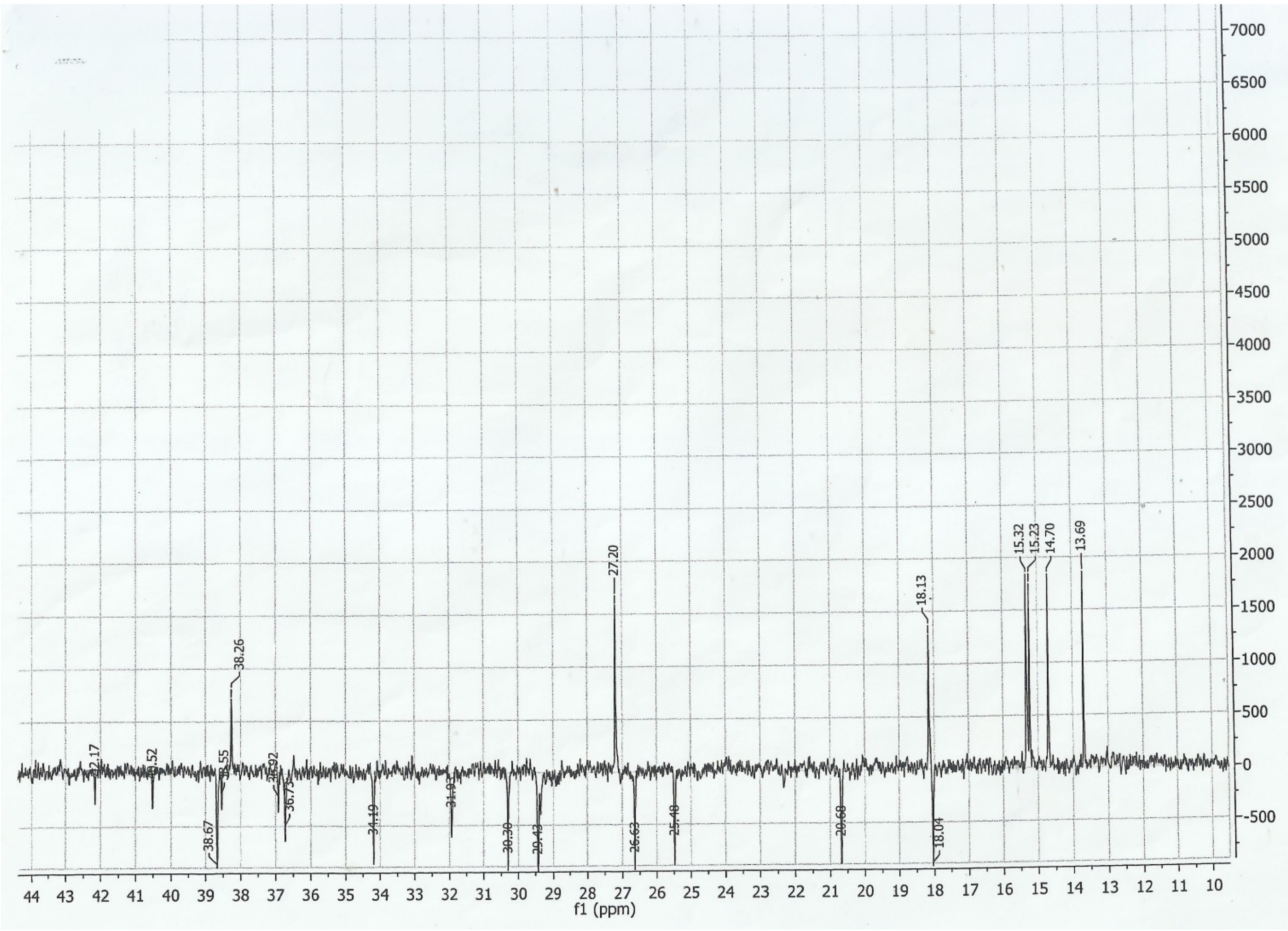

Figure S4. ^{13}C-NMR (DEPT) spectrum of betulin.

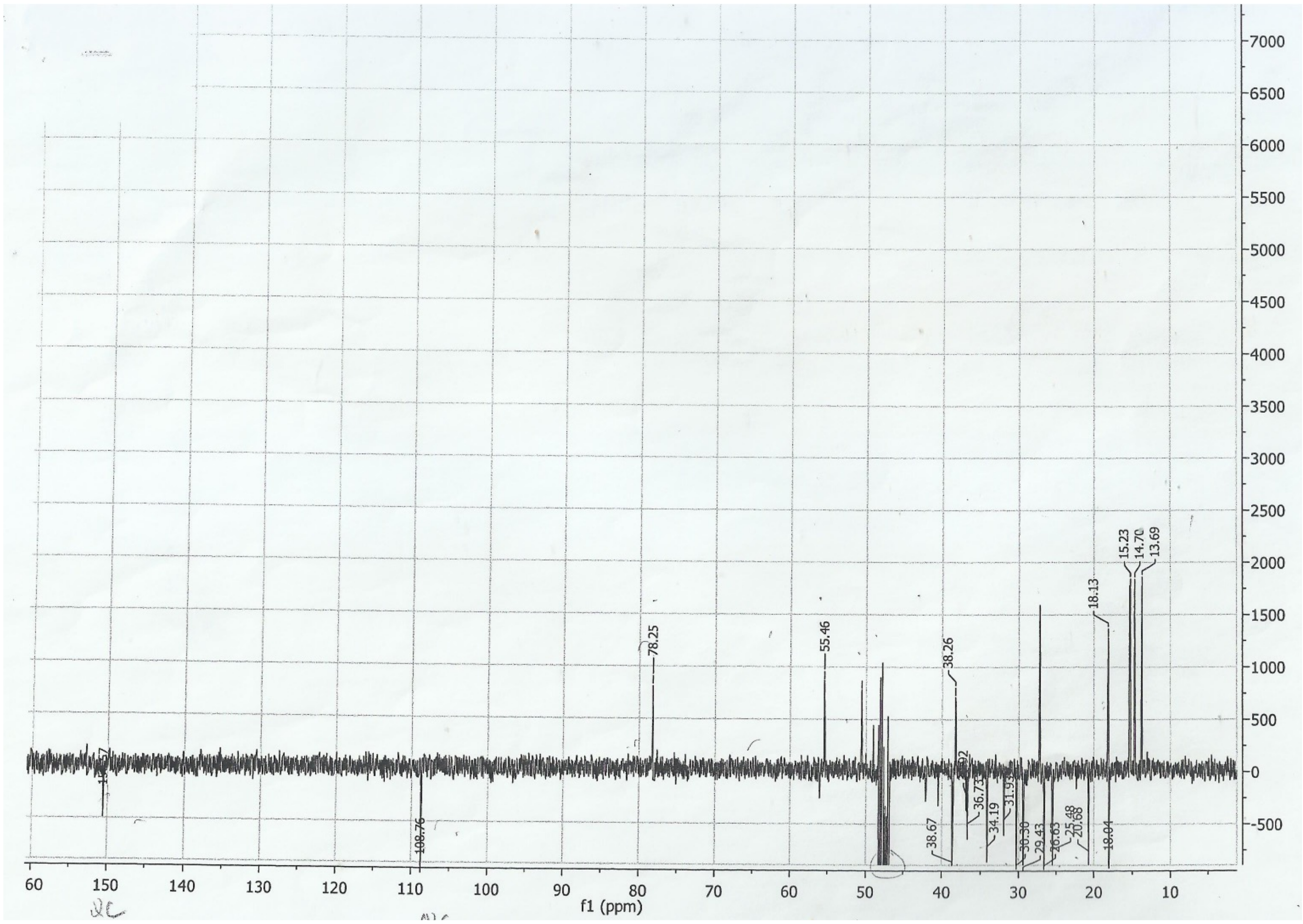

Figure S5. ^{13}C-NMR (DEPT) spectrum of betulin (Expansion 1).

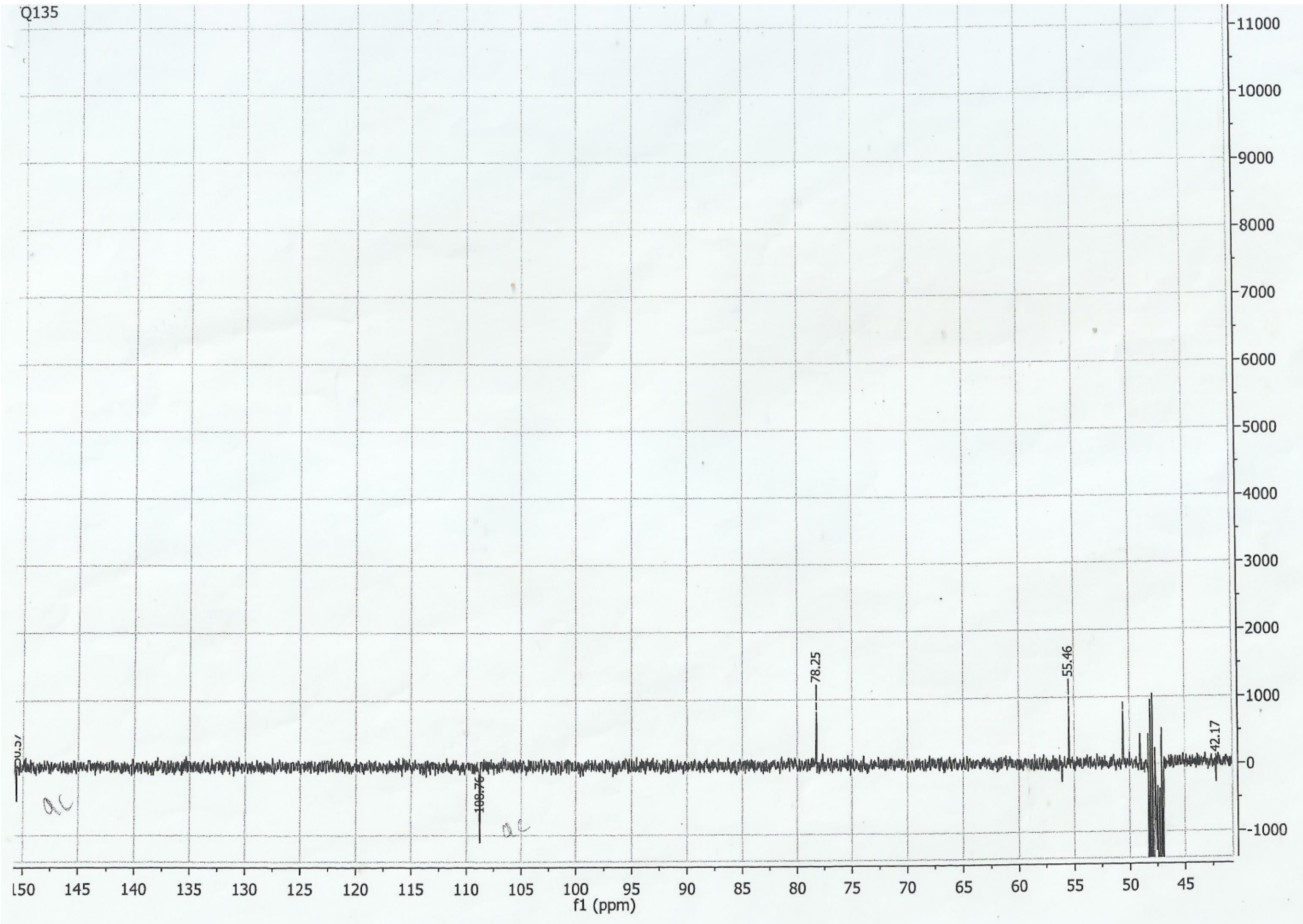

Figure S6. ^{13}C-NMR (DEPT) spectrum of betulin (Expansion 2).

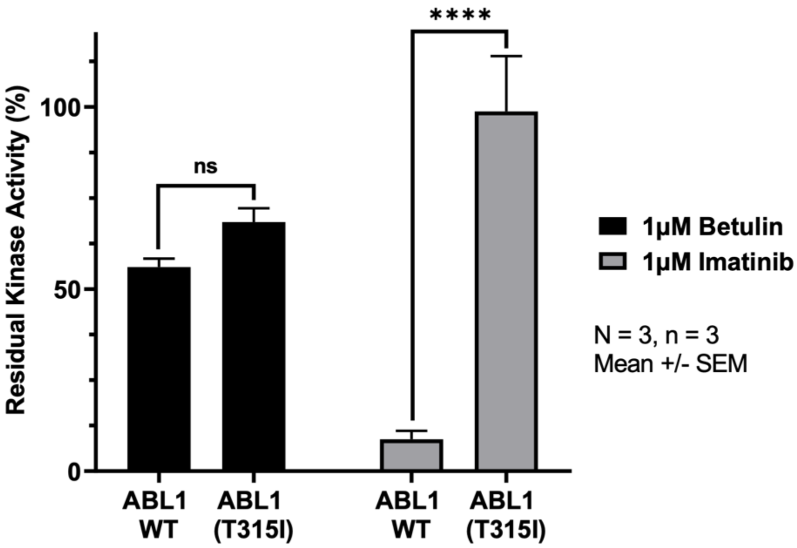

Figure S7. Effect of the T315I ABL1 kinase domain mutation on the inhibition by betulin. We selected 1 µM as the fixed concentration of inhibitor, since this value approximates the IC_{50} value for betulin against wild-type (WT) kinase. We measured inhibition of mutant and WT ABL1 kinase activities by betulin and by imatinib, using the ADP-Glo luminescent assay. ATP concentration used in the kinase assays was 10 µM. Data are mean (N=3, n=3) ± SEM expressed as % of residual kinase activity, normalized to the DMSO control. ns, not significant, ****: $p < 0.0001$.

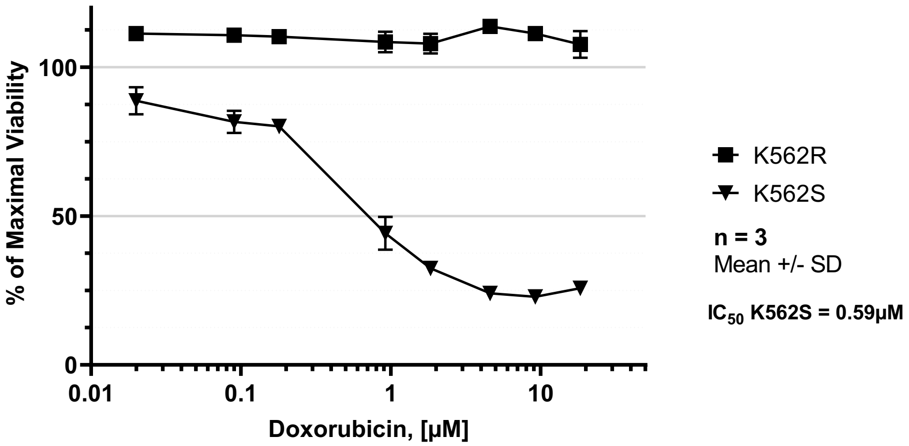

Figure S8. *In vitro* effect of doxorubicin on the cell viability of K562 chronic myelogenous leukemia (CML) cells. The viability of K562S and K562R CML cell lines, which are sensitive (S) and resistant (R) to treatment with doxorubicin, respectively, was studied using the MTS assay. Cell viability was measured following a 48-hour exposure to increasing doses of doxorubicin. The IC_{50} value was determined from the dose-response curve using GraphPad PRISM Software. Data are expressed as mean (n=3) ± SD, of % maximal viability (normalized to cells treated with a DMSO vehicle alone).

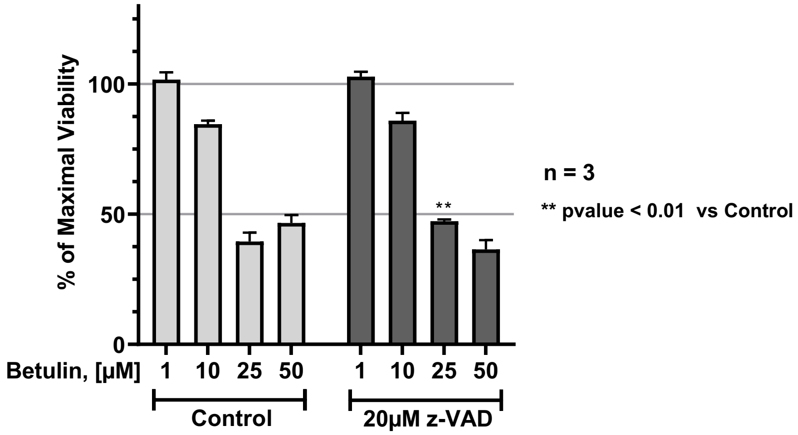

Figure S9. The effect of treatment with pan caspase inhibitor z-VAD-fmk on the phenotype induced by betulin. The viability of K562S CML cell line was studied using the MTS assay. Cell viability was measured following a 48-hour exposure to increasing doses of betulin (1 - 50 µM, as shown), either alone or treated simultaneously with 20 µM z-VAD-fmk. Data are expressed as mean (n=3) ± SD, of % maximal viability (normalized to viability of cells treated with DMSO vehicle alone). ** $p < 0.01$ vs control.

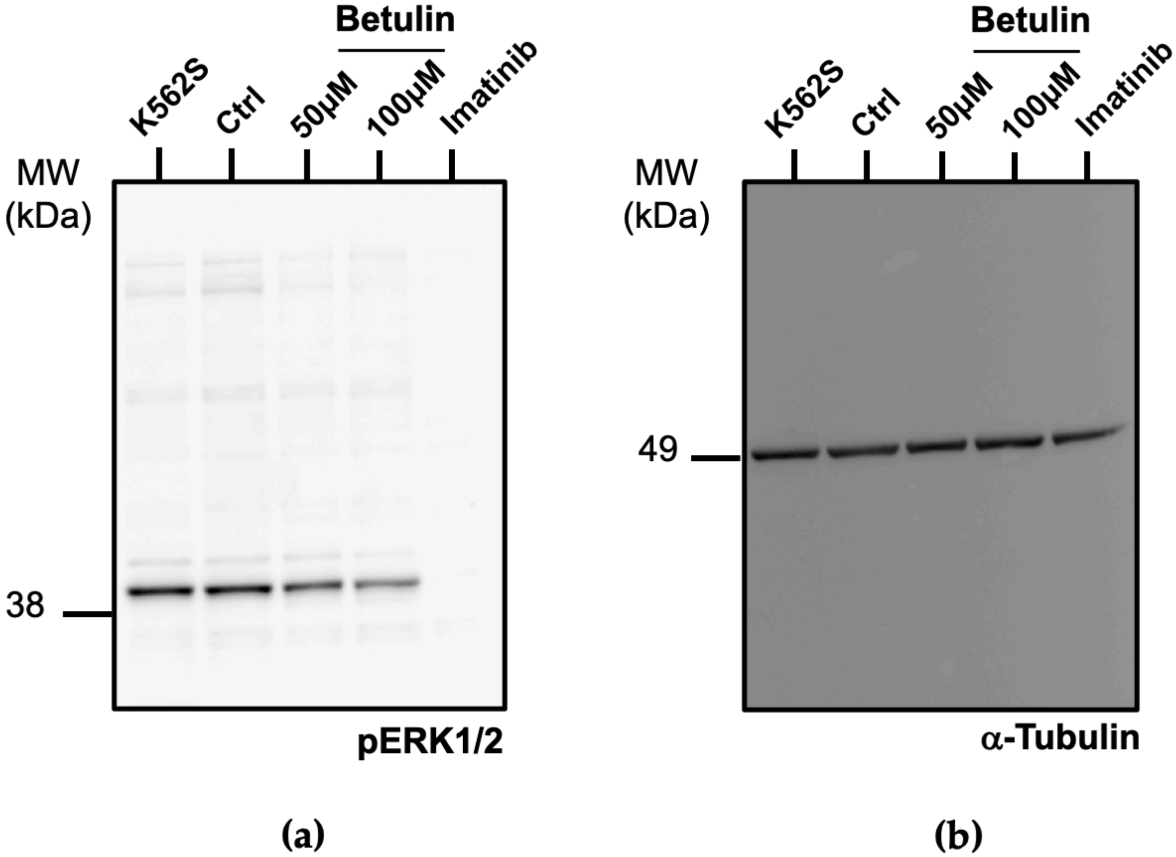

Figure S10. Effects of betulin on extracellular signal-regulated kinase (ERK) signaling. K562S CML cells were either untreated (K562S) or treated with 1 % DMSO, 50 or 100 μM of betulin or 20 μM of imatinib mesylate for 6 hours and immunoblot analysis was conducted as described in the Methods section. Extracts of K562S cells were analyzed by SDS-PAGE followed by Western blotting with antibodies directed against phospho-ERK1/2 (Thr202/Tyr204) (a) and α-Tubulin loading control (b).
